# Clinical importance of the informed consent process in breast surgery

**DOI:** 10.11604/pamj.2022.41.188.28872

**Published:** 2022-03-08

**Authors:** Sherif Monib

**Affiliations:** 1 St Albans Hospital breast unit, West Hertfordshire Hospitals NHS Trust, London, United Kingdom

**Keywords:** Consent, breast, reconstruction

## Abstract

Breast reconstruction operations have been on the rise over the last two decades; with recent advances in medical practice, different options of breast reconstruction have been readily available. However, it is challenging for patients to grasp the knowledge and digest differences, advantages, and disadvantages of each and every procedure to be able to choose the best procedure for them. Following the Thefaut and Montgomery cases, clinicians are obliged to make sure that all the required information is presented to the patients in a structured manner and suitable environment to aid them in deciding the best procedure for them and guiding them through the informed consent process.

## Opinion

The consent process is the patient's agreement to proceed with physical examination, investigation, medical/surgical treatment, organ donation or enrolment in a clinical trial proposed by the healthcare provider. Consent is not a one-off event, but is an ongoing process. The conscious patient can withhold or withdraw consent before or during treatment [1]. The principle of consent has always been there, as there has been evidence that a similar process was used by the ancient Egyptians, the Greeks and the Romans [2]. In 500 BC, the Hippocratic Oath advised clinicians to share the information with the patients to achieve better care [3].

The informed consent term was first used by attorney Pace E in a medical malpractice United States court case in 1957 [4]. In modern medical practice, informed consent is an ethical concept that is codified in the law. For consent to be valid, it must be voluntary, informed, and the person consenting must have the capacity to make the decision. The consent can be presumed, implied, verbal or written [5]. As patients have the right to determine what happens to their bodies, performing a procedure on a patient without valid informed consent is a form of battery and can lead to losing the medical registration and criminal charges. Hence, obtaining consent for a procedure is one of the core competencies for graduates expected by the General Medical Council [6].

A professionally conducted consent process ensures that clinicians are prompted to consider the evidence base concerning the proposed treatment and its alternatives. The patients are appropriately informed, their autonomy respected, and their expectations managed [7]. Fulfilling ethical and legal requirements for informed consent can sometimes be challenging due to issues like language barriers. Following the Thefaut and Montgomery cases where the court ruled that clinicians must ensure that patients are aware of all risks associated with any recommended treatment, clinicians have been urgently urged to update their consent processes [8].

Over the last two decades, a significant increase in breast reconstructions has been noted, with a 29% increase from 2000 to 2018 [9]. In addition, with the improvement of breast implants and relatively shorter operative and recovery time, there has been an international trend of increased implant-based reconstruction compared to autologous reconstruction [10]. Therefore, clinicians should tailor their practice to make sure they meet the ethical and lawful medical practice requirement, achieve optimum patient satisfaction, and decrease the number of breast reconstruction-related litigation. Hence, I strongly advise my colleagues to consider discussing and documenting these points before proceeding with breast reconstruction.

**General information:** include documenting the patient's height, weight, BMI, smoking status, pre-existing body dysmorphia or breast asymmetry (consider attaching preoperative photo). Also, discuss different reconstruction options, scar placement, operative and recovery time, and follow up.

**Implant-based reconstruction:** the general information section should include different types of implants (silicone/saline or combined expanders-anatomical/round-fixed volume or adjustable), the plain of implant placement (pre pectoral/sub pectoral), as well as the possible use of a mesh (Acellular Dermal Matrix (ADM) or synthetic mesh) to cover the lower pole. In addition to highlighting the need for other operations in future such as pocket adjustment, port removal, implant exchange, fat grafting, and contralateral symmetrising reduction. The agreed procedure which the patient is willing to proceed with needs to be clearly documented.

The possible complications' section should include general complications such as bleeding, collection, infection, pain, scar formation, delayed healing, alteration of sensation or reoperation, cardiac and pulmonary complications, venous thrombosis related complications, allergic reactions, lymphoedema and unsatisfactory results and its physical and psychological impact. Plus, implant-based reconstruction associated complications as the increased risk of infection, increased risk of implant loss following radiotherapy, shape and size miss-match, asymmetry, contour irregularity due to wrinkling and rippling, capsular contracture, calcification- implant displacement, implant failure, silicone gel bleed, silicone laden lymph nodes, and implant extrusion, chest wall irregularities, immune system diseases and unknown risks, Breast Implant-Associated Anaplastic Large Cell Lymphoma (BIA-ALCL).

**Autologous reconstruction:** the general information section should include different types of autologous reconstruction (pedicled/free flap). In addition, the agreed operation that the patient is willing to proceed with must be documented. Possible complications' section should include general complications as bleeding, collection, infection, pain, scar formation, delayed healing, alteration of sensation or reoperation, cardiac and pulmonary complications, venous thrombosis related complications, allergic reactions, lymphoedema and unsatisfactory results and its physical and psychological impact. Plus, autologous reconstruction specific complications such as weakness or the donor area, hernia formation, asymmetry, infection, delayed healing, fat necrosis and scar formation, complete or partial flap loss. Based on my practice and previous literature, patient satisfaction is clearly linked to their expectations, hence following good medical practice through a valid consent process leads to better patient outcomes.

**Limitations:** my opinion was based on my practice and available literature, which I am sure if more international collaborative work is carried out, we will come up with more robust data.
